# Primary renal Ewing’s sarcoma in an adult: an enigma

**DOI:** 10.1515/iss-2020-0022

**Published:** 2021-01-07

**Authors:** Suvraraj Das, Gaurav Aggarwal, Sujoy Gupta, Divya Midha

**Affiliations:** Surgical Oncology, Tata Medical Center, Newtown, Kolkata, West Bengal, India; Department of Uro-Oncology, Tata Medical Center, Newtown, Kolkata, West Bengal, India; Department of Onco-Pathology, Tata Medical Center, Newtown, Kolkata, West Bengal, India

**Keywords:** adjuvant chemotherapy, extraskeletal Ewings sarcoma, primary renal Ewings sarcoma, radical nephrectomy

## Abstract

**Objectives:**

Extraskeletal Ewing’s sarcoma is a rarity, with a renal primary in an adult, being even rarer. There is no consensus on the optimal imaging modality, as well as best therapeutic option, making them an enigma for clinicians.

**Case presentation:**

We report the case of a 34-year-old lady, a known case of invasive lobular carcinoma of the left breast (ER,PR positive, Her2neu negative), having completed treatment in 2017, wherein, on an ultrasound evaluation for left flank pain, was incidentally found to have a left renal mass. A CT scan corroborated with the ultrasound, with an additional Level 1, left renal vein thrombus. She underwent an open left radical nephrectomy with renal vein thrombectomy. Histopathology of the resected tumor revealed features of Ewing’s sarcoma of the kidney, confirmed by Fluorescent In Situ Hybridisation (FISH) and Immunohistochemistry (IHC).

**Conclusion:**

Primary renal Ewing’s sarcoma in an adult is a rare occurrence, with no characteristic imaging features, and no universally accepted guideline based management protocols. Akin with standard Ewings sarcoma treatment strategies, a margin negative- radical nephrectomy with adjuvant chemotherapy, seems the most apt treatment strategy.

## Introduction

Ewing’s sarcoma constitutes a group of malignant round small cell tumours of primitive neuroectodermal origin with the most common sites being bone and soft tissues, particularly in children and young adults. Extraskeletal Ewing’s Sarcoma is a rarity, with a renal primary in an adult, being even more sparse.

Literature suggests that Ewing’s sarcoma of the kidney (ESK) is aggressive and has a propensity for rapid clinical progression and metastasis. It is characterised by a balanced chromosomal translocation t(11;22)(q24;q12) which results in the production of the EWS/Friend leukaemia virus integration 1 (FLI-1) fusion gene. The treatment of choice remains radical nephrectomy followed by combination chemotherapy with or without adjuvant radiotherapy [1]. Despite this, the overall prognosis and survival of Ewing’s sarcoma of the kidney remains grim.

## Case report

A 32 year old lady, a known and treated case of invasive lobular carcinoma of left breast in 2017, on follow up, presented with left flank pain since 2 months. She gave no history of haematuria or fever. She was evaluated with an Ultrasound-abdomen, which showed the presence of a left renal SOL (Size: 17×14×11 cm).

**Figure 1:**
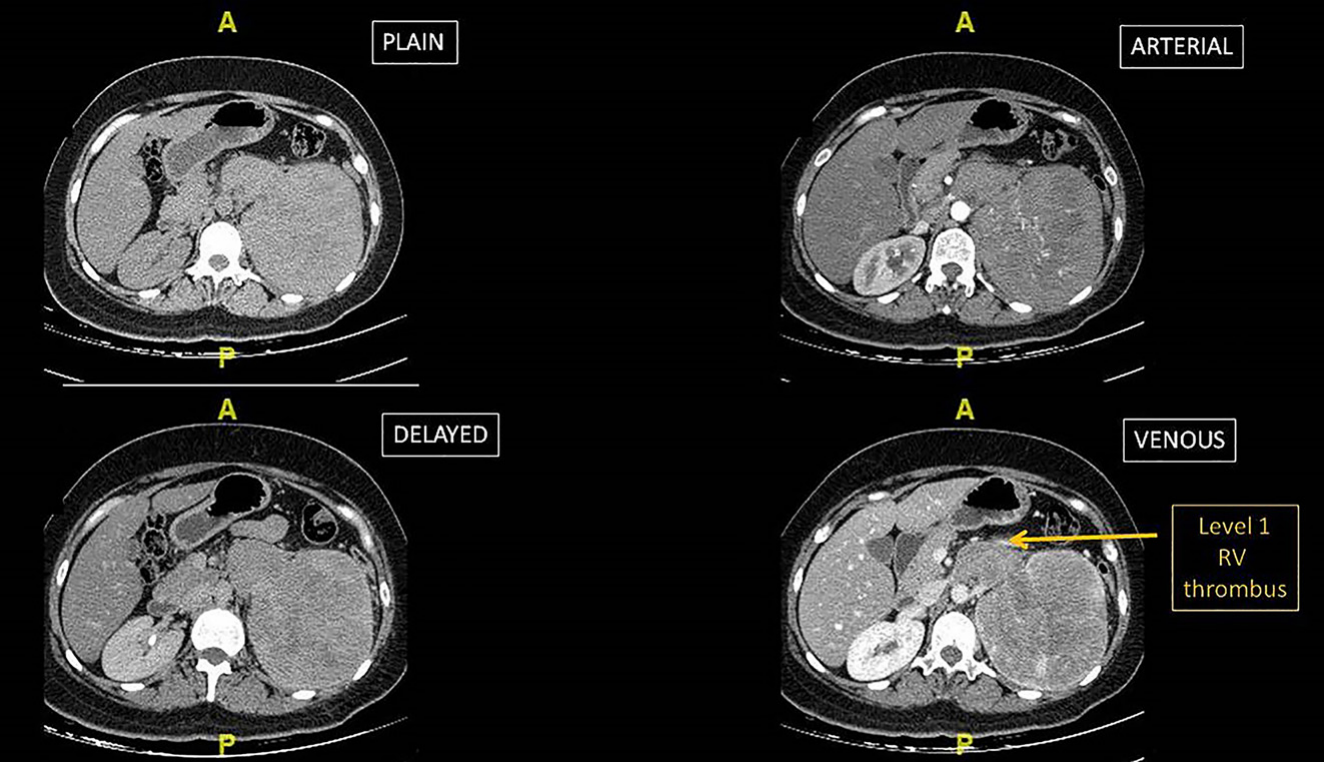
CT scan abdomen- different phases, showing the Level 1 renal vein thrombus.

A CT scan abdomen confirmed the ultrasound findings, with an additional Level 1, left renal vein thrombus (Figure 1). CT thorax and other biochemical tests were normal.

With a working diagnosis of left renal cell carcinoma, she underwent an open left radical nephrectomy with left renal vein thrombectomy and retroperitoneal lymph node dissection.

The procedure and post-operative course was uneventful, and she was discharged on day 5, without event.

**Figure 2:**
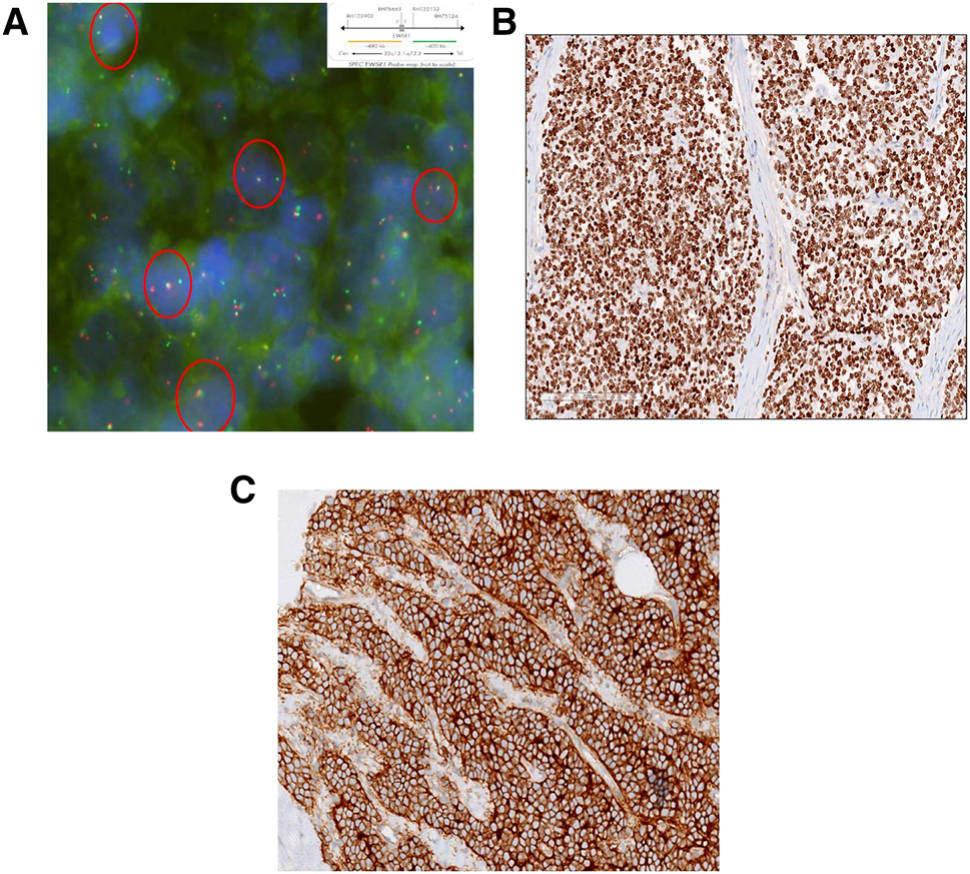
(A) Positive fusion fluorescence in situ hybridization (FISH) results (1 yellow fusion signal and 1 pair of split red and green signals in most nuclei), (B) Immunohistochemistry (IHC) image, NKX 2.2 positive, (C) Immunohistochemistry (IHC) image, CD 99 positive.

The post-operative histopathology report, however, confirmed the diagnosis of an Unifocal Ewing’s sarcoma of greatest dimension, 16 cm with lymphovascular (LVI) and perineural invasion (PNI) present. Resected margins were free of tumour. 2 of 9 perihilar lymph nodes were involved by tumour and Pre, para and interaortocaval lymph nodes were negative for malignancy. FISH was positive for Ewing sarcoma breakpoint region 1 (EWSR1) (Figure 2A). On Immunohistochemistry, the tumour cells were diffusely positive for NKX2.2 (Figure 2B) and CD99 (Figure 2C) while negative for PAX8 and GATA3.

A bone scan done subsequently revealed no skeletal metastasis. The final histopathology report was discussed in our multidisciplinary tumour board meeting, and she was started on adjuvant chemotherapy, Vincristine + Adriamycin + Cyclophosphamide (VAC) regimen alternating with Ifosphamide and Etoposide (IE).

## Discussion

Ewing’s sarcoma constitutes a group of malignant round small cell tumours of primitive neuroectodermal origin with the most common sites being bone and soft tissues, particularly in children and young adults. The average age at diagnosis is usually 30.4 years with a male to female ratio of 60:1.

Primary renal Ewings sarcoma is a rarity, with clinical and radiological features mimicking other common renal tumours. An accurate diagnosis is usually possible post-operatively with a combination of FISH and immunochemistry [2]. A characteristic t(11;22)(q24;q12) is highly specific for PNET/Ewing’s sarcoma and differentiates it from neuroblastoma and adult variety of Wilm’s tumour.

Renal Ewing’s behaves more aggressively than at other sites. It has strong propensity for local recurrence, early metastasis to lymph nodes, lungs liver and bone [3]. Due to the rarity of primary renal Ewing’s sarcoma/PNET, a standardized treatment protocol has not been established.

In general, Ewing’s sarcoma treatment uses a multimodality approach with debulking surgery (radical nephrectomy), chemotherapy with or without radiotherapy to the renal bed as the possible options. Even after multimodality treatment, prognosis remains poor with an overall 5-year disease free survival of 45–55% [4]. The combination chemotherapy reportedly used in Ewing’s sarcoma includes VDC/VAC or vincristine, Adriamycin, and cyclophosphamide alternating with Ifosphamide and Etoposide.

Adjuvant radiotherapy can be offered in case of incomplete resection, positive resection margins, or recurrence, which was not so in our case.

Follow-ups with laboratory and imaging tests are essential to assess recurrence and metastasis.

## Conclusion

Primary renal Ewing’s sarcoma is an aggressive tumour, particularly in adults, with a high propensity of metastases. With no specific imaging characteristics to delineate the same, a multimodality treatment approach mandating a margin negative- radical nephrectomy with adjuvant combination chemotherapy is recommended, in sync with the management of Ewing’s tumors at other body sites. Literature reports a poor overall prognosis, despite all forms of therapy, for this enigmatic condition.
