# Supplementary material for: Primary renal Ewing’s sarcoma in an adult: an enigma
Source: Innov Surg Sci. 2021 Jan 7;5(3-4):111–3. doi: 10.1515/iss-2020-0022 (PMC7790175; doi:10.1515/iss-2020-0022)
Supplement: Supplementary file 1 [file iss-05-20200022-s001.pdf]

Reviewer Assessment

Gaurav Aggarwal et. al: Primary Renal Ewing's Sarcoma in an Adult : An Enigma

Reviewers' Comments to Original Submission

Reviewer 1: anonymous

Date received: 16-Sep-2020
Reviewer recommendation: **Accept in present form**
Reviewer overall scoring: **Medium**

Assessment Form scores: 5 = High/Yes; 3 = Medium/Adequate; 1 = Low

Is the subject area appropriate for the journal	5				
Does the title clearly reflect the paper's content?	5				
Does the abstract clearly reflect the paper's content		4			
Do the keywords clearly reflect the paper's content?		4			
Does the introduction present the problem clearly?		4			
Are the results/ conclusions justified?		4			
How comprehensive and up-to-date is the subject matter presented?			3		
How adequate is the data presentation?			3		
Are units and terminology used correctly?		4			
Is the number of cases adequate?					1
Are the experimental methods/ clinical studies adequate?		4			
Is the length appropriate in relation to the content?		4			
Does the reader get new insights from the article?					1
Please rate the practical significance.				2	
Please rate the accuracy of methods.		4			
Please rate the statistical evaluation and quality control.			3		
Please rate the appropriateness of the figures and tables.			3		
Please rate the appropriateness of the references.			3		
Please evaluate the writing style and use of language.		4			
Please judge the overall scientific quality of the manuscript.				2	
Are the methods used worthy of reproduction in greater deal?	No				
Would you be willing to review a revision of this manuscript?	No				

Comments to author: This is a well crafted and clear case report with limited benefit for real-world clinical problems of other practitioners.

Reviewer 2: anonymous

Date received: 29-Oct-2020
Reviewer recommendation: **Accept in present form**
Reviewer overall scoring: **Medium**

Assessment Form scores: 5 = High/Yes; 3 = Medium/Adequate; 1 = Low

Is the subject area appropriate for the journal		4			
Does the title clearly reflect the paper's content?		4			
Does the abstract clearly reflect the paper's content		4			
Do the keywords clearly reflect the paper's content?		4			
Does the introduction present the problem clearly?		4			
Are the results/ conclusions justified?		4			
How comprehensive and up-to-date is the subject matter presented?			3		
How adequate is the data presentation?			3		
Are units and terminology used correctly?	5				
Is the number of cases adequate?					1
Are the experimental methods/ clinical studies adequate?			3		
Is the length appropriate in relation to the content?		4			
Does the reader get new insights from the article?			3		
Please rate the practical significance.			3		
Please rate the accuracy of methods.		4			
Please rate the statistical evaluation and quality control.			3		
Please rate the appropriateness of the figures and tables.		4			
Please rate the appropriateness of the references.		4			
Please evaluate the writing style and use of language.			3		
Please judge the overall scientific quality of the manuscript.			3		
Are the methods used worthy of reproduction in greater deal?	No				
Would you be willing to review a revision of this manuscript?	No				

Comments to author: This is a well-written case report, that can be published as presented.
